# Binge-like Alcohol Exposure in Adolescence: Behavioural, Neuroendocrine and Molecular Evidence of Abnormal Neuroplasticity… and Return

**DOI:** 10.3390/biomedicines9091161

**Published:** 2021-09-04

**Authors:** Anna Brancato, Valentina Castelli, Gianluca Lavanco, Giuseppe Tringali, Vincenzo Micale, Martin Kuchar, Cesare D’Amico, Giuseppe Pizzolanti, Salvatore Feo, Carla Cannizzaro

**Affiliations:** 1 Department of Health Promotion, Mother and Child Care, Internal Medicine and Medical Specialties of Excellence “G. D’Alessandro”, University of Palermo, 90127 Palermo, Italy; anna.brancato@unipa.it (A.B.); giuseppe.pizzolanti@unipa.it (G.P.); 2 Department of Biomedicine, Neurosciences and Advanced Diagnostics, University of Palermo, 90127 Palermo, Italy; valentina.castelli02@unipa.it; 3 INSERM U1215, NeuroCentre Magendie, 330300 Bordeaux, France; gianlucalavanco@gmail.com; 4 University of Bordeaux, 33300 Bordeaux, France; 5 Department of Biomedical and Biotechnological Sciences, University of Catania, 95123 Catania, Italy; vincenzomicale@inwind.it; 6 Pharmacology Section, Department of Health Care Surveillance and Bioethics, Università Cattolica del Sacro Cuore, Largo F. Vito 1, 00168 Rome, Italy; giuseppe.tringali@unicatt.it; 7 Department of Chemistry of Natural Compounds, University of Chemistry and Technology, 166 28 Prague, Czech Republic; kuchara@vscht.cz; 8 Department of Biological, Chemical and Pharmaceutical Sciences and Technologies, University of Palermo, 90128 Palermo, Italy; cesare.damico@unipa.it (C.D.); salvatore.feo@unipa.it (S.F.)

**Keywords:** binge alcohol drinking, adolescence, nucleus accumbens, cannabidiol

## Abstract

Binge alcohol consumption among adolescents affects the developing neural networks underpinning reward and stress processing in the nucleus accumbens (NAc). This study explores in rats the long-lasting effects of early intermittent exposure to intoxicating alcohol levels at adolescence, on: (1) the response to natural positive stimuli and inescapable stress; (2) stress-axis functionality; and (3) dopaminergic and glutamatergic neuroadaptation in the NAc. We also assess the potential effects of the non-intoxicating phytocannabinoid cannabidiol, to counteract (or reverse) the development of detrimental consequences of binge-like alcohol exposure. Our results show that adolescent binge-like alcohol exposure alters the sensitivity to positive stimuli, exerts social and novelty-triggered anxiety-like behaviour, and passive stress-coping during early and prolonged withdrawal. In addition, serum corticosterone and hypothalamic and NAc corticotropin-releasing hormone levels progressively increase during withdrawal. Besides, NAc tyrosine hydroxylase levels increase at late withdrawal, while the expression of dopamine transporter, D1 and D2 receptors is dynamically altered during binge and withdrawal. Furthermore, the expression of markers of excitatory postsynaptic signaling—PSD95; Homer-1 and -2 and the activity-regulated spine-morphing proteins Arc, LIM Kinase 1 and FOXP1—increase at late withdrawal. Notably, subchronic cannabidiol, during withdrawal, attenuates social- and novelty-induced aversion and passive stress-coping and rectifies the hyper-responsive stress axis and NAc dopamine and glutamate-related neuroplasticity. Overall, the exposure to binge-like alcohol levels in adolescent rats makes the NAc, during withdrawal, a *locus minoris resistentiae* as a result of perturbations in neuroplasticity and in stress-axis homeostasis. Cannabidiol holds a promising potential for increasing behavioural, neuroendocrine and molecular resilience against binge-like alcohol harmful effects.

## 1. Introduction

Heavy binge drinking is highly prevalent among high-school and college students in western countries [1]. Compared to adults, adolescents are less responsive to many effects of alcohol intoxication and withdrawal (motor impairment, sedation hangover, anxiety), but are more sensitive to positive effects of alcohol like euphoria and the facilitation of social interaction [2,3,4]. This combination of sensitivities to alcohol facilitates alcohol drinking by adolescents at well above binge levels [5], defined by the National Institute on Alcohol Abuse and Alcoholism as an intake of 4–5 drinks per session producing blood alcohol concentrations of at least 0.08 g/dL [6]. The mesolimbic dopamine (DA) system, consisting of the ventral tegmental area (VTA) and the nucleus accumbens (NAc), as well as the associated limbic structures, is involved in the reward and reinforcing effects of alcohol [7,8]. Alcohol stimulates the DA-ergic mesolimbic system by acutely increasing the firing rate of VTA DA neurones [9,10,11] and DA release in the NAc [12,13,14]. Interestingly, withdrawal after chronic ethanol exposure decreases VTA cell-firing in the NAc for several days after removal, inducing a profound disarrangement in the electrophysiological properties and the anatomic architecture of the synaptic triad in adult rats [15,16]. The accumbal synaptic triad is thought to allow for DA-mediated gating of cortico-striatal glutamatergic synaptic transmission [17], playing a regulatory role in spine maintenance and stability in medium spiny neurones (MSNs), the main population of intrinsic GABAergic neurones. Indeed, a subtle balance between DA levels and glutamate release critically regulates synaptic plasticity associated with alcohol abuse and withdrawal [18].

Notably, alcohol may modify the developmental reorganization of the mesocorticolimbic system, which is a typical process of adolescence. Specifically, the NAc is a hub where alcohol interacts with pathways related to affective state control and reward processing, modifying the trajectories of neurodevelopmental and synaptic remodeling that, in turn, can impair behavioural responses [19,20]. Indeed, developmental rearrangement during adolescence results in an imbalance between a weak excitatory cortical control and a supersensitive DA-ergic signaling in the mesocorticolimbic pathway [21,22], thus creating a window of increased vulnerability for excessive alcohol consumption [23]. In detail, the glutamatergic and DA-ergic asset is dynamic during adolescence, when the density of NMDA [24] and DA receptors typically increases [25,26,27], spontaneous firing rates of DA neurones are higher [28] as well as DA turnover [29], and DA transporter density [30].

Moreover, during adolescence, marked changes occur in brain circuits implicated in responsiveness to stress and emotional stimuli [31], which are profoundly sensitive to alcohol exposure [32]. Whereas indisputably adolescents exhibit heightened social interaction [33], they also display elevated reactivity to discrete stressful cues [34]. This suggests a potential developmental sensitivity of younger populations to prolonged alcohol exposure, as suggested by the clinical evidence showing that alcohol-abusing adolescents exhibit enhanced stress reactivity, anxiety and social avoidance [35].

Similarities have been found between human adolescents and adolescents of various mammalian species in terms of developmental history and behavioural changes, as well as neural and hormonal alterations [29,36]. This provides reasonable justification for the use of animal models for the assessment of anxiety-related behaviour under social circumstances during adolescence. The social interaction test has been used extensively for the assessment of social anxiety-like behaviour in laboratory rodents [37]. It was found that reductions in social preference and/or social investigation may reflect an increase in emotional state that results in dysfunctional social reward/aversion processing [38,39]. Notably, the response to novelty and motivated behaviour are strongly interconnected. Hyponeophagia in the novelty-suppressed feeding test (NSFT) is an example: in this case the inhibition of feeding is caused by the exposure to a novel arena, and the conflict appears between the anxiogenic environment and hunger-induced behaviour [40].

The development of lasting negative emotional states following chronic alcohol consumption has been studied for decades in rodents [41]. However, little is known about the onset of negative affective state and abnormal behavioural reactivity in rats subjected to intoxicating alcohol levels every other day, along a paradigm of intermittent binge-like exposure during adolescence. On this basis, we aimed at assessing social anxiety-like behaviour, the response to novelty and the sensitivity to natural positive stimuli, and coping strategies against inescapable stress, together with corticosterone secretion and hypothalamic and extrahypotalamic CRH expression, at different stages of the drinking paradigm: during binge days (BD), at withdrawal day 1 (WD1) and withdrawal day 10 (WD10). Moreover, in order to investigate to what extent a disarray of the synaptic triad in the NAc could play a role in the behaviours observed, on the basis of previous study of this group [15,16], we assessed: (1) DA signaling, by measuring presynaptic tyrosine hydroxylase (TH) expression and DA transporter (DAT) density by immunofluorescence, D1 and D2 receptor mRNA in the MSN in the NAc; (2) indices of excitatory postsynaptic dynamics, through the expression of excitatory scaffold markers (.e., post synaptic density protein 95 (PSD95) and Homer 1 and 2 expression); (3) actin polymerization activity-regulated spine-morphing proteins (i.e., activity-regulated cytoskeleton-associated protein (Arc), and Lim Domain Kinase 1 [LIM Kinase 1]); and (4) MSN maturity upgrade by Forkhead box p1 (FOXP1) expression.

Given that recent evidence suggests that nonpsychotropic phytocannabinoid cannabidiol (CBD) exerts favorable effects on stress-related conditions [42,43] and addictive behaviour [44,45,46,47] we assessed if repeated CBD treatment during withdrawal may reverse the behavioural and molecular alterations induced by intermittent alcohol exposure.

## 2. Materials and Methods

### 2.1. Animals

Wistar rats from Envigo (Italy) arrived on postnatal day (PND) 21, were housed in pairs (unless differently indicated) in standard polycarbonate cages with standard bedding and maintained at 22 ± 2 °C with 55 ± 5% humidity on a 12 h light/dark cycle (lights on 08:00 AM). Laboratory rodent chow (Mucedola, Italy) and tap water were available ad libitum, apart from overnight food restriction before the novelty suppressed feeding test. Procedures were approved by the Italian Ministry of Health (1119/2016-PR), in adherence with the current Italian regulation (D.L. 26/2014) and the European directive (2010/63/EU) on laboratory animals’ care and use. Every effort was made to minimize the number of animals used and their suffering.

### 2.2. Drugs

Alcohol (96%; Carlo Erba Reagenti, Milan, Italy) was dissolved in tap water at 25% *v*/*v*. Cannabidiol (2-[(1R, 6R)-6-Isopropenyl-3-methylcyclohex-2-en-1-yl]-5pentylbenzene-1,3-diol) (CBD), extracted by the Forensic Laboratory of Biologically Active Substances of the University of Chemistry and Technology of Prague, Czech Republic (purity (NMR) > 99%) [48], was dissolved in a vehicle of ethanol (1%), Tween 80 (1%), saline and. immediately afterward, administered intraperitoneally (i.p.) at the dose of 60 mg/kg [46].

### 2.3. Binge Alcohol Exposure

Rats were exposed to alcohol in an intermittent binge-like paradigm *per os*, three days a week, every other day, during adolescence (PND 35–54, [49]) for a total of nine exposures. Daily, 25% alcohol was prepared from alcohol 96° (Carlo Erba Reagenti, Italy) diluted with tap water, and administered at the dosage of 3.5 g/kg [50]. Controls were given an isovolumetric amount of tap water on the same exposure days. Rats were gently administered alcohol (or water) by introducing the calculated amount of solution in the rat’s mouth thought a laboratory pipette. This procedure aimed to decrease the distress of gavage in adolescent rats and employ the common administration route of alcohol consumption in humans with accurate dosing [51].

### 2.4. Behavioural Procedures

**Figure 1:**
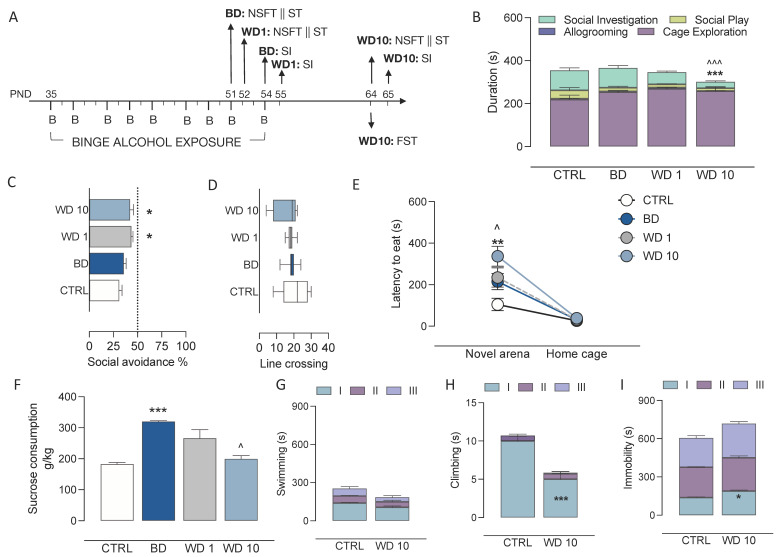
Binge-like alcohol exposure during adolescence disrupts reward/aversion behaviour during withdrawal. (**A**) Rats exposed to a binge-like alcohol drinking paradigm during adolescence did not show social behaviour alterations at BD, whereas they displayed (**B**) decreased duration of social investigation at WD10, as compared with CTRL (*** *p* < 0.001) and BD (^^^ *p* < 0.001) and (**C**) increased social anxiety-like behaviour, in terms of social avoidance %, at WD1 and WD10, when compared with CTRL (* p < 0.05) (**D**) in the presence of no difference in the number of line crossings, as a measure of locomotor activity. In addition, (**E**) rats exposed to a binge-like alcohol drinking paradigm during adolescence did not show novelty-induced anxiety-like behaviour in the novelty-suppressed feeding test at BD, since no difference in latency to eat in the novel arena was measured with respect to CTRL, while, at WD10, they show increased latency to eat when compared with CTRL (** *p* < 0.01) and BD (^ *p* < 0.05), who showed no difference in latency to eat in their home cage. On the other hand, (**F**) at BD rats showed increased sensitivity to natural reward in the sucrose-preference test as compared with CTRL rats (*** *p* < 0.001), which significantly decreased at WD10 (^ *p* < 0.05). (**G**–**I**) When tested for coping under forced-swim stress, Rats exposed a to binge-like alcohol drinking during adolescence showed (**G**) similar swimming duration as CTRL rats, however, WD10 rats displayed a passive coping strategy withing the first 5 min of the test, wherein we observed (**H**) decreased climbing and (**I**) increased immobility duration, as compared with the CTRL counterpart. BD: binge day; WD1: withdrawal day 1; WD10: withdrawal day 10; CTRL: water-exposed control group; NSFT: novelty-suppressed feeding test; ST: sucrose-consumption test; SI: social-interaction test; FST: forced-swim test. Each bar and each circle represent the mean of *n* = 7 rats; error bars indicate SEM.

Every effort was made to minimize the number of animals used and their suffering. Behavioural testing occurred in the three experimental groups at different intervals from the binge-level alcohol exposure: we tested on binge days, for the group BD (45 min after the alcohol administration; a novelty suppressed feeding test (NSFT) and sucrose consumption test (ST) was given after the 8th alcohol administration (PND 51); a social interaction test (SI) was given after the 9th administration (PND 54); at withdrawal day 1, for the group WD1, (1 day after the alcohol administration); NSFT and ST were performed, 1 day after the alcohol 8th administration (PND 52); SI was performed 1 day after the 9th administration (PND 55) and, starting from WD10, was also performed for the group WD10 (NSFT and ST, at PND 64; SI at PND 65) (Figure 1A). In order to minimize the distress of repeated testing and the exposure to stressful procedures, a second cohort of rats at WD10 was assessed for stress-coping in the modified forced swim test (FST). Testing of the CTRL rats occurred at PND 64. CBD, or vehicle (ethanol [1%]) and Tween 80 [1%], saline) were administered during withdrawal on PND 55, 57, 59, 61, 63, thus the last administration occurred 24 h before behavioural testing All testing procedures were conducted between 9:00 AM and 1:00 PM under dim light (15–20 lux). For tissue collection, rats of the first cohort were sacrificed after the social interaction test.

#### 2.4.1. Novelty-Suppressed Feeding Test (NSFT)

Rats were food-restricted overnight before testing and, on the test day, they were habituated to the testing room for 1 h. Under dim light conditions, rats were then placed into a plastic box 50 × 50 × 20 cm with bedding. A single pellet of food was positioned in the center of the box. Rats were then placed in the corner of the box, and the latency to eat was scored for as long as 10 min during testing [40]. Rats were then immediately transferred to their home cage in standard lighting conditions, and their latency to eat was recorded.

#### 2.4.2. Sucrose-Consumption Test (ST)

Rats were individually housed, and one of their two water bottles was replaced with a 1% sucrose bottle for 24 h. The bottles were then weighed, and sucrose consumption was calculated by dividing the weight of sucrose solution consumed by the rat’s body weight (g/kg) [52].

#### 2.4.3. Social Interaction Test (SI)

The testing apparatus (45 × 45 × 30 cm) was partially divided into two equally sized compartments by a white plexiglass partition, which allowed the movement of the rats between them [50]. On the test day, rats were taken from their home cage and placed individually in the testing apparatus for a 2-min habituation. A social partner of the same age and sex was then introduced for a 10-min test period. Social partners were always unfamiliar with the experimental animal and were experimentally and drug naive. During the test session, the behaviour of the rats was recorded by a video camera for later scoring. The frequency and duration of social investigation, allogrooming, social play and cage exploration (rearing and moving in the cage) were analysed from video recordings by a trained experimenter blind to the experimental conditions. Social investigation was defined as the sniffing of any part of the body of the partner. Social-play scoring included play fighting, such as pinning, pouching, nape-attacking, and following and chasing, wherein the experimental animal rapidly pursues the partner. Aggressive behaviour was not displayed by any of the rats. In addition, the time spent in the empty cage compartment (nonsocial), and the time spent in the same cage compartment of the social partner (social) were measured. Social avoidance was calculated by the percentage of time spent in the nonsocial compartment; in addition, motor behaviour was measured in terms of number of partition crossings during the 2-min habituation.

#### 2.4.4. Forced Swim Test (FST)

The modified forced swim test here employed was previously described in [53]. In this single-session test, rats were placed individually in clear cylinders (40 cm high, 18 cm inside diameter) filled with 5–6 L of clean water at 22–23 °C, for 15 min. The sessions were videotaped for subsequent analysis. The 15-min session was divided into three separate time bins (5 min each), and the durations of swimming, climbing and immobility was recoded. Swimming behaviour was defined as movement throughout the swim chamber; climbing behaviour consisted of upward-directed movements of the forepaws along the side of the cylinder; immobility was assigned when no additional activity was observed other than that required to keep the rat’s head above the water. Cumulative scores through the entire session were kept.

### 2.5. Tissue Collection

Rats of the first cohort were killed and trunk blood samples were collected in the early afternoon (1:00–3:00 PM). Serum was prepared according to standard protocols and kept at −20 °C until the time of assay. Brains were rapidly removed to ice and divided into two sagittal halves by employing a brain matrix. One half was immediately immersed in cold 4% paraformaldehyde for 24 h fixation at 4 °C, while the second half was sliced into 1 mm-thick coronal sections, on ice, and NAc samples were rapidly dissected. Tissue samples were flash-frozen in dry ice and stored at −80 °C until subsequent analysis. Brain hemispheres were counterbalanced among experiments.

### 2.6. Blood Alcohol Concentration Measurements

In order to assess blood alcohol concentration, trunk blood was sampled 1 h after the last binge alcohol exposure. Blood alcohol concentration was quantified in the serum by employing a commercially available colorimetric assay kit (STA-620; Cell Biolabs, Inc., San Diego, CA, USA) according to the protocol supplied by the manufacturer.

### 2.7. Corticosterone Determination

Serum corticosterone levels (CORT, ng/mL) were measured using a commercially available ELISA kit (Demeditec Diagnostics GmbH, Kiel, Germany), according to the manufacturers’ instructions [54].

### 2.8. RNA Extraction and qRT-PCR

RNA was isolated using homogenization in Trizol (Invitrogen) followed by chloroform layer separation. The clear RNA layer was then processed (RNAeasy MicroKit, QIAGEN, Germantown, MD, USA) and analysed with NanoDrop (ND-1000 Spectrophotometer, Thermo Scientific, Wilmington, DE, USA). A total of 500 ng of RNA was then reverse transcribed to cDNA (SuperScript IV Reverse Trascriptase, Invitrogen). cDNA was diluted to 500 uL, and 3 uL were used for each reaction. The reaction mixture consisted of PowerUp SYBR Green (Master Mix (2X), Applied Biosystems) (12.5 μL), forward and reverse primers (2 μL), water (8 μL), and the cDNA template. Samples were then heated to 95 °C for 10 min followed by 40 cycles of 95 °C for 15 s, 60 °C for 1 min, 95 °C for 15 s, 60 °C for 30 s and 95 °C for 15 s. Analysis was performed using the delta–delta C(t) method. Primers employed are indicated in Table 1.

### 2.9. Immunofluorescence Experiments

Fixed brains were coronally sectioned at a thickness of 35 μm using a microtome (Campden Instruments, Loughborough, UK). Serial sections were collected through the rostrocaudal dimensions (every sixth slice) and stored at 4 °C in 0.02% sodium azide in phosphate-buffered saline (PBS) until immunofluorescence staining. Brain sections, including the NAc (2.20 mm to 1.60 mm from bregma [55]) or the paraventricular hypothalamic nucleus (PVN) (−1.4 mm to −1.9 mm from bregma [55]) were washed in PBS for 30 min and incubated in blocking solution (3% normal goat or donkey serum + 0.3% Triton X-100 in PBS) for 2 h, at room temperature, under gentle shaking. Sections were then incubated in primary antibody solution for 24 h at 4 °C under gentle shaking (3% normal serum, 0.3% Tween-20 in PBS), with rat anti-DAT (1:100; Santa Cruz Biotechnology, Dallas, TX, USA); mouse anti-TH (1:500; Santa Cruz Biotechnology, Dallas, TX, USA) or goat anti-PSD95 (1:1000, Abcam). For CRH immunofluorescent staining, sections were incubated with sheep corticotropin-releasing hormone antibody (NB110-81721, Novus, USA) at a dilution of 1:200 for 72 h, at 4 °C, under gentle shaking. Sections were washed in PBS for 1 h, incubated in a secondary antibody solution for 2 h under gentle shaking (goat anti-rat Alexa Fluor 488, 1:400; goat anti-mouse Alexa Fluor 594, 1:400; donkey anti-goat Cy2, 1:400; donkey anti-sheep Cy3, 1:400, Jackson ImmunoResearch, West Grove, PA, USA). After 1 h washing in PBS, slices were briefly incubated with DAPI (1 mg/mL), mounted onto a Superfrost^®^ Plus (Thermo Scientific, Fisher Scientific GmbH, Schwerte, Germany) and coverslipped using Vectashield^®^‘s HardSet TM Antifade mounting medium. Images were acquired using an epifluorescence microscope (Meji Techno, Saitama, Japan) and the software Deltapix Insight, Smorum, Denmark. Positive immunofluorescence was quantified for each image as integrated density over threshold using Image J and verified by a trained experimenter. For each NAc section, the value was calculated as the mean of the core and shell quantifications [56].

### 2.10. Data Calibration and Statistical Analysis

When data exhibited normality and equal variance, the difference between groups was determined by employing either one-way, two-way or three-way analysis of variance (ANOVA), including “binge alcohol exposure” as the between-subject factor, and “environment” or “session” as the repeated-measure factor, or “CBD treatment” as the within-subject factor. The Bonferroni post hoc test was employed, when necessary. If data did not show normal distribution or equal variance, a Kruskal–Wallis ANOVA on ranks and Dunn’s post hoc test were performed. Data are reported as mean ± SEM. In radar graph representation, measures were standardized to a common scale by z-transformation with respect to the reference condition as follows: standardized data = (raw data − average control group)/standard deviation of the control group. Hence, standardized values are cantered at zero for the reference group, whereas the mean values for the treatment group are rescaled relative to this [57]. Principal component analysis (PCA) was used to examine patterns of intercorrelations between the variables studied in cohort 1 [58]. The principal components produced by the orthogonal linear transformation of PCA are linear combinations of the original measures, on a new coordinate system, reflecting independent characteristics or dimensions underlying the correlation matrix [59]. The first principal component or vector is associated to the largest possible variance, whereas the second represents most of the remaining variation and so forth, with the first three components generally explaining most of the variance. The loading-factor value of each measure indicates its importance for the principal component [59]. In the present study, principal components that together account for 75% of the total explained variance were highlighted. Here, the original datasets of each individual rat, containing 20 variables, including 7 parameters related to behaviour (social investigation, social play, social anxiety, locomotor activity, latency to eat in a novel environment and in their home cage, sucrose consumption), 3 variables related to neuroendocrine stress response (CORT, hypothalamic and accumbal CRH), 4 variables related to pre- and postsynaptic DA signaling (TH, DAT, D1R, D2R), 6 markers of postsynaptic MSNs signaling (PSD95, HOM1, HOM2, Arc, LIMK, FOXP1) were standardized using z-scores and analysed to obtain their correlation matrix and PCAs as previously described [58]. Statistical analysis was performed using Prism v. 9 (Graphpad) and statistical significance was set at alpha = 0.05.

## 3. Results

### 3.1. Alcohol Binging during Adolescence Jeopardizes Affective Behaviour

Adolescent rats were exposed to binge-like alcohol administration in an intermittent drinking paradigm. In our experimental conditions, when rats were sampled on the last BD, 1 h after binge alcohol exposure, they displayed a BAC of 193 ± 19 mg/dL, indicating that binge-like alcohol levels (>80 mg/dL) were reached in this study. The effects of binge-like alcohol exposure during adolescence on affective behaviour, including the social, emotional and hedonic dimensions, were evaluated at BD, WD1 and WD10 (Figure 1A). Our data show that binge-like alcohol exposure during adolescence discretely altered social behaviour in the modified social interaction test (Figure 1B–D). In particular, we observed a significant effect on social investigation (F(3,24) = 11.1, *p* < 0.0001), with a decrease at WD10 with respect to BD (t = 4.8, *p* = 0.0004) and CTRL (t = 4.81, *p* = 0.0003); no differences were observed in the durations of allogrooming (F(3,24) = 2.30, *p* = 0.1032), social play (Kruskal–Wallis test: *p* = 0.0808) or cage exploration (F(3,24) = 2.13, *p* = 0.1223) (Figure 1B). In addition, binge-like alcohol exposure during adolescence increased anxiety-like behaviour under social circumstances during withdrawal (F(3,24)= 4.56, *p* = 0.0115). In particular, we observed a significant increase in the avoidance of the compartment occupied by a conspecific social partner at WD1 (t = 3.23, df = 24, *p* = 0.0215) and WD10 (t = 2.89, df = 24, *p* = 0.0486) with respect to CTRL (Figure 1C). No significant effects on locomotor activity, in terms of number of barrier crossings, was observed (F(3, 24) = 0.982, *p* = 0.4177) (Figure 1D). When rats were assessed in the NSFT, data analysis of their latency to eat, in the novel arena and their home cage, indicates a significant main effect of binge-like alcohol exposure during adolescence (F(3,48) = 5.75, *p* = 0.0019), of novel environment (F(1,48) = 83.2, *p* < 0.001) and of the interaction between the two statistical factors (F(3,48) = 4.65, *p* = 0.0062). Bonferroni post hoc tests show that latency to eat in the novel arena, at BD, was not different in comparison with CTRL rats (t = 2.64, df = 48, *p* = 0.0676). However, a significant increase was observed at WD1 (t = 3.11, df = 48, *p* = 0.0187) and WD10 (t =5.56, df = 48, *p* < 0.001) with respect to CTRL rats. Moreover, rats at WD10 displayed increased latency to eat in the novel arena with respect to BD (t = 2.92, df = 48, *p* = 0.0320). Measures of latency to eat in the home cage showed no differences (*p* > 0.999) (Figure 1E). When assessed for their response to a natural reward, rats preferred, in all instances, the sucrose solution to water. However, while water consumption did not differ between the groups (CTRL: 4.78 ± 0.40 mL; BD: 5.14 ± 1.15 mL; WD1: 3.85 ± 0.32 mL; WD10: 5.00 ± 0.70 mL; F(3,24) =0.6378, *p* = 0.5980), binge-like alcohol exposure during adolescence altered sucrose consumption (Kruskal–Wallis test: *p* = 0.0016), with a significant increase at BD as compared with CTRL rats (*p* = 0.0021), whereas sucrose consumption decreased at WD10 with respect to BD (*p* = 0.0280) (Figure 1F). On the other hand, the analysis of data from the FST shows that binge-like alcohol exposure during adolescence modified rat’s stress-coping. When swimming duration across the three-time bins was analysed, a trend for binge-like alcohol exposure during adolescence was observed (F(1,12) = 3.936, *p* = 0.0706) (Figure 1G). In addition, during the first 5-min time bin of the test, WD10 rats displayed decreased climbing (F(1,12) = 20.52, *p* = 0.0007; Bonferroni post hoc: t= 8.489, df = 36.00; *p* < 0.001) (Figure 1H) and increased immobility (F(1,12) = 8.264, *p* = 0.0140; Bonferroni post hoc: t= 2.995 df = 36.00; *p* = 0.0148) (Figure 1I) with respect to CTRL rats.

### 3.2. Binge-like Alcohol Exposure during Adolescence Perturbs the Neuroendocrine Stress Response

**Figure 2:**
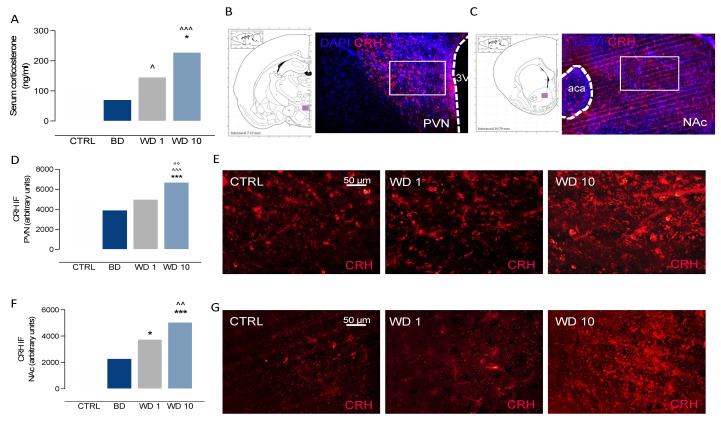
Binge-like alcohol exposure during adolescence perturbs the neuroendocrine stress response during withdrawal. Rats exposed a to binge-like alcohol drinking paradigm during adolescence (**A**) did not show corticosterone serum alterations on BD, whereas they displayed a progressive increase of corticosterone during WD1, when compared with BD (^ *p* < 0.05), and at WD10, with respect to CTRL (* *p* < 0.05) and BD (^^^ *p* < 0.001). When neuroendocrine stress response was assessed in terms of (**B**) hypothalamic and (**C**) extrahypothalamic CRH immunofluorescence, we found (**D**,**E**) a significant increase in CRH immunofluorescence in the PVN at WD10, when compared with CTRL (*** *p* < 0.001), BD (^^^ *p* < 0.001) and WD1 (°° *p* < 0.01) and (**F**,**G**) a significant increase in CRH immunofluorescence in the NAc at WD1, with respect to CTRL rats (* *p* < 0.05), and at WD10 with respect to CTRL (*** *p* < 0.001) and BD (^^ *p* < 0.01) rats. PVN: paraventricular hypothalamic nucleus; 3V: third ventricle; NAc: nucleus accumbens; aca anterior commissure. BD: binge day; WD1: withdrawal day 1; WD10: withdrawal day 10; CTRL: water-exposed control group. Each bar represents the mean of *n* = 7 rats; error bars indicate SEM.

Binge-like alcohol exposure during adolescence altered the responsiveness of the HPA axis, in terms of serum CORT levels (Kruskal–Wallis test *p* = 0.0002). Indeed, CORT levels increased during withdrawal, with higher levels at WD1 than at BD (Dunn’s multiple comparisons test: *p* = 0.0341), and significantly increased at WD10 when compared with BD (*p* = 0.0001) and CTRL rats (*p* = 0.0416) (Figure 2A). When the neuroendocrine stress response was assessed in terms of hypothalamic (Figure 2B) and extrahypothalamic (Figure 2C) CRH immunofluorescence, we observed that binge-like alcohol exposure during adolescence increased CRH immunofluorescence in the PVN during withdrawal (F(3, 24) = 18.5, *p* < 0.0001). In detail, CRH levels significantly increased at WD10 with respect to CTRL (t = 5.47, df = 24, *p* < 0.0001), BD (t = 7.12, df = 24, *p* < 0.0001) and WD1 (t = 4.39, df = 24, *p*< 0.0001) (Figure 2D,E). Moreover, Rats exposed a to binge-like alcohol drinking during adolescence showed altered CRH levels in the NAc (Kruskal–Wallis test: *p* < 0.001), where CRH immunostaining was significantly increased at WD1, with respect to CTRL (Dunn’s post hoc test: *p* = 0.0133), and at WD10 with respect to CTRL (*p* < 0.0001) and BD (*p* = 0.0054) rats (Figure 2F,G).

### 3.3. Binging on Alcohol during Adolescence Alters Neuroplasticity in the NAc during Withdrawal

**Figure 3:**
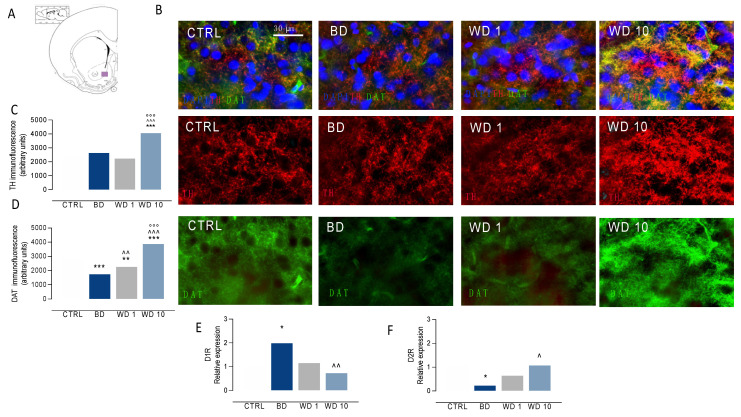
Binge-like alcohol exposure during adolescence alters DA signaling in the NAc. Rats exposed to binge-like alcohol drinking during adolescence show (**A**–**F**) altered pre- and postsynaptic markers of DA neurotransmission in the NAc. In particular, we observed (**B**,**C**) increased TH-positive immunofluorescence at WD10, when compared with CTRL (*** *p* < 0.001), BD (^^^ *p* < 0.001) and WD1 (°°° *p* < 0.001); on the other hand, we found (**B**,**D**) decreased DAT-positive levels at BD and WD1, when compared with CTRL (*** *p* < 0.001; ** *p* < 0.01), whereas DAT levels increase at WD10, with respect to CTRL (*** *p* < 0.001), BD (^^^ *p* < 0.001) and WD1 (°°° *p* < 0.001) groups. Opposite changes were observed in D1- and D2R expression levels (**E**,**F**). In detail, (**E**) D1R mRNA relative expression increased at BD with respect to the CTRL group (* *p* < 0.05), and decreased during withdrawal, with a significant decrease at WD10 when compared with BD (^^ *p* < 0.01). On the other hand, (**F**) D2R mRNA relative expression significantly decreased at BD when compared with CTRL rats (* *p* < 0.05) and significantly increased at WD10, with respect to BD (^ *p* < 0.05). NAc: nucleus accumbens; TH: tyrosine hydroxylase; DAT: dopamine transporter; D1R: dopamine receptor 1; D2R: dopamine receptor 2; BD: binge day; WD1: withdrawal day 1; WD10: withdrawal day 10; CTRL: water-exposed control group. Each bar represents the mean of *n* = 7 rats; error bars indicate SEM. All expression values were normalized to control mean.

**Figure 4:**
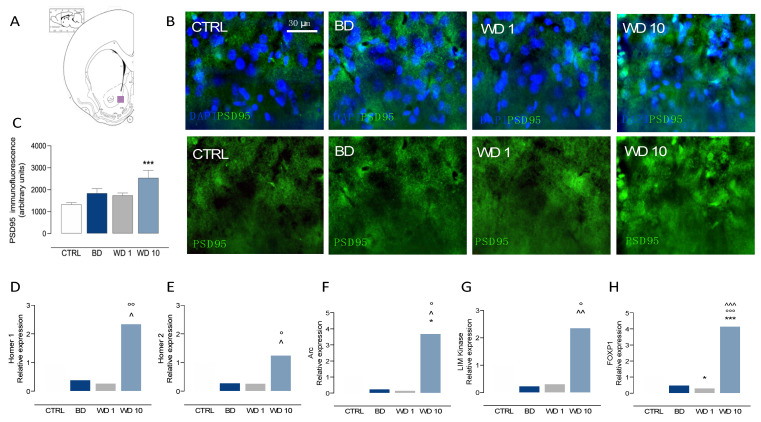
Binge-like alcohol exposure during adolescence perturbs post-synaptic excitatory signaling in the NAc. (**A**–**H**) Rats exposed to binge-like alcohol drinking during adolescence show altered levels of post-synaptic markers of glutamate neurotransmission and activity-regulated proteins at late withdrawal in the NAc. In particular, we observed (**B**,**C**) increased PSD95-positive immunofluorescence at WD10, when compared with CTRL (*** *p* < 0.001) group; in addition, we found (**D**) increased Homer 1 mRNA relative expression at WD10 when compared to BD (^ *p* < 0.05) and WD1 (°° *p* < 0.01), and (**E**) increased Homer 2 relative expression at WD10 with respect BD (^ *p* < 0.05) and WD1 (° *p* < 0.05) groups. Besides this, (**F**–**H**) a significant increase of activity-regulated proteins expression was observed at WD10, as to (**F**) increased Arc relative expression at WD10, with respect to CTRL (* *p* < 0.05), BD (^ *p* < 0.05) and WD1 (° *p* < 0.05); (**G**) increased LIM Kinase 1 relative expression at WD10, with respect to BD (*p* < 0.01) and WD1 (° *p* < 0.05); (H) increased FOXP1 relative expression when compared with CTRL (*** *p* < 0.001), BD (^^^ *p* < 0.001) and WD1 (°°° *p* < 0.001). NAc: nucleus accumbens; PSD95: post-synaptic density protein 95; Arc: activity-regulated cytoskeleton-associated protein; LIM Kinase: LIM Kinase 1; BD: binge day; WD1: withdrawal day 1; WD10: withdrawal day 10; CTRL: water-exposed control group. Each bar represents the mean of *n* = 7 rats; error bars indicate SEM. All expression values were normalized to control mean.

When we examined the effects of binge-like alcohol drinking during adolescence on DA signaling in the NAc (Figure 3A–F), our data show a significant main effect of binge-like alcohol exposure on TH-positive immunofluorescence (F(3,24) = 31.3, *p* < 0.0001), with increased levels at WD10 in comparison with CTRL (t = 7.87, df = 24, *p* < 0.0001), BD (t = 6.72, df = 24, *p* < 0.001) and WD1 (t = 8.65, df = 24, *p* < 0.001) (Figure 3B,C). In addition, data analysis highlights a significant main effect on DAT-positive immunofluorescence (F(3,24) = 92.0, *p* < 0.0001), with decreased levels at BD (t = 8.21, df = 24, *p* < 0.001) and WD1 (t = 4.39, df = 24, *p* = 0.012) with respect to CTRL rats. On the other hand, DAT-positive immunofluorescence increased during withdrawal, with higher levels at WD1 than at BD (t = 3.82, df = 24, *p* = 0.005), and at WD10 when compared with CTRL (t = 7.59, df = 24, *p* < 0.001), BD (t = 15.8, df = 24, *p* < 0.001) and WD1 (t = 12.0, df = 24, *p* < 0.001) (Figure 3B,D). In addition, binge-like alcohol exposure during adolescence modified gene expression relevant to DA signaling in the NAc. In detail, binge-like alcohol exposure affected D1 receptor expression (F(3,24) = 5.318, *p* = 0.0059), with increased levels at BD with respect to CTRL (t = 3.005, df = 24, *p* = 0.0306) and a significant decrease at WD10 with respect to BD (t = 3.766, df = 24, *p* = 0.0047) (Figure 3E). Furthermore, binge-like alcohol during adolescence modified D2 receptor expression (F(3,24) = 4.279, *p* = 0.0149), with decreased levels at BD when compared with the CTRL group (t = 3.088, df = 24, *p* = 0.0251) and a significant increase at WD10 with respect to BD (t = 3.004, df = 24, *p* = 0.0307) (Figure 3F). On the other hand, binge-like alcohol exposure during adolescence altered postsynaptic excitatory signaling in the NAc (Figure 4A–H). The analysis of PSD95 immunofluorescence shows significant differences among the different time points considered (F(3,24) = 5.81, *p* = 0.0039), with a significant increase at WD10 when compared with CTRL (t = 4.10, df = 24, *p* = 0.0024) (Figure 4A–C). In addition, we observed altered Homer 1 expression (Kruskal–Wallis test: *p* = 0.0084), with increased levels at WD10 with respect to BD (Dunn’s post hoc test: *p* = 0.0479) and to WD1 (Dunn’s post hoc test: *p* = 0.009) groups (Figure 4D). Similarly, Homer 2 expression in the NAc was significantly altered during withdrawal from binge-like alcohol exposure (Kruskal–Wallis test: *p* = 0.00187), with increased levels at WD10 when compared to BD (Dunn’s post hoc test: *p* = 0.0459) and WD1 (Dunn’s post hoc test: *p* = 0.0309) (Figure 4E). Furthermore, we observed significant effects on Arc expression (Kruskal–Wallis test: *p* = 0.0047), with increased levels at WD10 with respect to CTRL (Dunn’s post hoc test: *p* = 0.0148), BD (*p* = 0.0376) and WD1 (*p* = 0.0148) (Figure 4F). As to LIM Kinase 1 expression, we observed a significant increase at WD10 when compared to BD (Kruskal–Wallis test: *p* = 0.0049; Dunn’s post hoc test: *p* = 0.0086) and WD1 (*p* = 0.0165) (Figure 4G). Interestingly, binge-like alcohol exposure during adolescence modified the expression of FOXP1 (F(3,24) = 104.4, *p* < 0.001), with decreased levels at WD1 with respect to CTRL rats (t = 3.168, df = 24, *p* = 0.0249), and increased expression at WD10, when compared with CTRL (t = 12.32, df = 24, *p* < 0.001), BD (t = 14.77, df = 24, *p* < 0.001) and WD1 (t = 15.49, df = 24, *p* < 0.001) groups (Figure 4H).

### 3.4. Affective and Emotional Behaviour in Binge-like Alcohol-Exposed Rats Correlates with Neuroendocrine Stress Response and Transcriptional Alterations in the NAc

**Figure 5:**
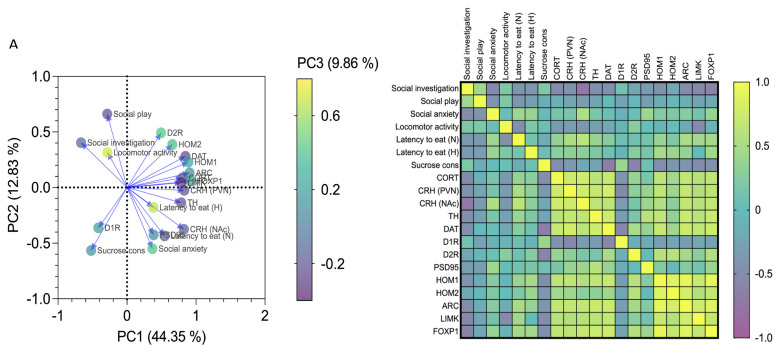
Affective and emotional behaviours in binge-like alcohol-exposed rats correlate with neuroendocrine stress response and transcriptional alterations in the NAc. (**A**) Loading-factor graph of principal component analysis shows how alterations of neuroendocrine stress response and protein expression at both presynaptic and postsynaptic levels in the NAc co-variate with affective end emotional behaviour observed in binge-like alcohol-exposed rats during adolescence. (**B**) The correlation matrix shows that altered affective and emotional behaviours correlate with neurobiological changes.

Alterations of the neuroendocrine stress response and protein expression at both prsynaptic and postsynaptic levels in the NAc may explain abnormal affective end emotional behaviour occurring in binge-like alcohol-exposed rats. Here we studied the relationship among 20 variables referring to social, emotional and affective behaviours, neuroendocrine stress response and markers of neuroplasticity in the NAc, by using PCA. When data from CTRL, BD, WD1 and WD10 rats were pooled, 67.05% of the overall variance was explained by the first three components. The main component, corresponding to 44.35% of the variance, was characterized by 15 of 20 variables on the positive side, with the exception of social investigation, social play, locomotor activity, sucrose consumption and D1 receptor expression (Figure 5A). The correlation matrix shows that social investigation was positively associated with social play (*p* = 0.019, r = 0.441), and negatively associated with social anxiety (*p* = 0.004, r = −0.528), latency to eat in the novel environment (*p* = 0.044, r = −0.383) and in the home cage (*p* = 0.047, r = −0.379), CORT (*p* = 0.005, r = −0.511), hypothalamic CRH (*p* = 0.004, r = −0.523) and accumbal CRH (*p* < 0.001, r= −0.740), TH (*p* = 0.014, r = −0.461), DAT (*p* = 0.013, r = −0.462), Homer1 (*p* = 0.015, r = −0.454), Arc (*p* = 0.006, r = −0.502), LIM Kinase 1 (*p* = 0.002, r = −0.556) and FOXP1 (*p* = 0.0007, r = −0.604). In addition, social play was positively associated with social investigation (*p* = 0.019, r = 0.441) and negatively associated with social anxiety (*p* = 0.028, r = −0.414) and latency to eat in the novel cage (*p* = 0.042, r = −0.387). As for social anxiety, it was positively associated with latency to eat in the novel arena (*p* = 0.028, r = 0.415), CRH- (*p* = 0.003, r = 0.537) and PSD95-positive immunofluorescence (*p* = 0.0259, r = 0.420), and FOXP1 expression (*p* = 0.0474, r = 0.463), and negatively associated to social investigation (*p* = 0.004, r= −0.528) and social play (*p* = 0.028, r= −0.414). On the other hand, latency to eat in the novel environment was positively correlated with social anxiety (*p* = 0.028, r = 0.415), hypothalamic CRH (*p* = 0.006, r = 0.507) and accumbal CRH (*p* = 0.0003, r= 0.634), TH (*p* = 0.048, r= 0.377), Homer 1 (*p* = 0.041, r = 0.388), Arc (*p* = 0.008, r = 0.493), LIMK1 (*p* = 0.020, r = 0.437) and FOXP1 expression (*p* = 0.0134, r = 0.462), in a positive trend with CORT (*p* = 0.067, r = 0.351), and negatively associated with social investigation (*p* = 0.044, r= −0.383), social play (*p* = 0.042, r= −0.387) and locomotor activity (*p* = 0.012, r= −0.470). Finally, sucrose consumption was not correlated with the behavioural variables analysed (*p* > 0.05), whereas it was positively associated with D1 receptor expression (*p* = 0.034, r = 0.403) and negatively associated with CORT (*p* = 0.011, r= −0.473), hypothalamic CRH (*p* = 0.0487, r= −0.376 and accumbal DAT-positive immunofluorescence (*p* = 0.0001, r = −0.664), and the expression of the D2 receptor (*p* = 0.001, r = −0.594), Homer 1 (*p* = 0.009, r = −0.484), Homer 2 (*p* = 0.036, r = −0.397), Arc (*p* = 0.028, r = −0.415), LIMK1 (*p* = 0.007, r = −0.499) and FOXP1 (*p* = 0.0145, r= −0.457) (Figure 5B).

### 3.5. CBD Mitigates Behavioural and Neuroendocrine Dysregulation, and NAc Maladaptive Neuroplasticity in Binge-Like Alcohol-Exposed Rats

**Figure 6:**
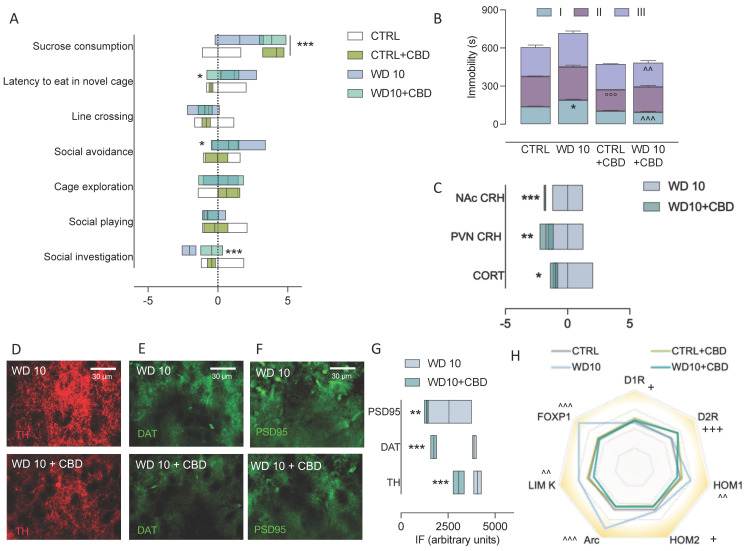
CBD rescues behavioural, neuroendocrine and NAc neuroplasticity maladaptations in binge-like alcohol-exposed rats. Sub-chronic CBD administration during withdrawal (**A**) increased social investigation (*** *p* < 0.001) and decreased both social avoidance (* *p* < 0.05) and latency to eat in the novel arena (* *p* < 0.05) in binge-like alcohol exposed rats; in addition, it increased sucrose consumption in both WD10 and CTRL rats (*** *p* < 0.001). Moreover (**B**) the passive coping observed at WD10 (* *p* < 0.05) was counteracted by CBD (^^^ *p* < 0.001), which also decreased immobility in CTRL rats within the second time bin (°°° *p* < 0.001) and in WD10 rats during the last time bin (^^ *p* < 0.01). As for HPA-axis alterations due to binge-like alcohol exposure during adolescence, (**C**) CBD decreased serum CORT levels (* *p* < 0.05) and CRH immunopositivity in the PVN (** *p* < 0.01) and the NAc (*p* < 0.001) in WD10 rats. In addition, CBD was able to counteract the effects of binge-like alcohol exposure during adolescence on neuroadaptations of DA and glutamate signaling in the NAc, with reference to (**D**,**G**) TH-positive immunofluorescence, (**E**,**G**) DAT immunopositivity and (**F**,**G**) PSD95 immunofluorescence, and in terms of gene the expression of (**H**) D1R (+ *p* < 0.05); D2R (+++ *p* < 0.001); HOM1 (^^ *p* < 0.01); HOM2 (+ *p* < 0.05); Arc (^^^ *p* < 0.001), LIM Kinase 1 (^^ *p* < 0.01); FOXP1 (^^^ *p* < 0.001). CBD: cannabidiol; WD10: withdrawal day 10; CTRL: water-exposed control group; PVN paraventricular hypothalamic nucleus; NAc: nucleus accumbens; CORT: corticosterone; CRH: corticotropin releasing hormone; TH: tyrosine hydroxylase; DAT: dopamine transporter; PSD95: post synaptic density protein 95; D1R: dopamine receptor 1; D2R: dopamine receptor 2; HOM1: Homer 1; HOM2: Homer 2; Arc: activity regulated cytoskeleton-associated protein; LIM K: LIM domain Kinase 1; FOXP1: Forkhead box P1. Each box represents the mean (line) and the range values of *n* = 7 rats; each bar represents the mean of *n* = 7 rats, and error bars indicate SEM. Unless differently indicated, boxes and lines in radar graph represent standardized values, cantered at zero for the CTRL group.

When administered sub-chronically during withdrawal, CBD ameliorated the affective alterations occurring in rats exposed to binge-like alcohol drinking during adolescence (Figure 6A,B). The analysis of social investigation in the modified social interaction test shows a significant effect of binge-like alcohol exposure (F(1,24) = 17.71, *p* = 0.0003), of CBD (F(1,24) = 5.574, *p* = 0.0267) and of the interaction between binge-like alcohol exposure and CBD (F(1,24) = 17.60, *p* = 0.0003). As a matter of fact, CBD did not modify social investigation in CTRL rats (t = 1.297, df = 24, *p* > 0.999) while it restored the social investigation deficit observed at WD10 (t = 4.636, df = 24.00, *p*= 0.0006) (Figure 6A). No significant effect of CBD or interaction was highlighted on data from social play (F(1,24) = 0.1245, *p* = 0.7272; F(1,24) = 0.2963, *p* = 0.5913) and cage exploration (F(1,24) = 0.01546, *p* = 0.9021; F(1,24) = 3.864, *p* = 0.0610). On the other hand, when social avoidance data were analysed, a significant effect of binge-like alcohol exposure (F(1,24) = 10.13, *p* = 0.004), CBD (F(1,24) = 5.896, *p* = 0.0230) and the rats’ interaction (F(1,24) = 5.364, *p* = 0.0294) was highlighted. CBD treatment did not modify social avoidance in control rats (t = 0.79, df = 24.00, *p* > 0.999) whereas it decreased the avoidance of the social compartment in WD10 rats (t = 3.355, df = 24.00, *p* = 0.0158) (Figure 6A). No significant effect on exploratory behaviour was observed (binge: F(1,24) = 2.027, *p* = 0.1674; CBD:F(1,24)= 4.114, *p* = 0.0538; interaction: F(1,24) = 1.110, *p* = 0.3025) (Figure 6B). In addition, a three-way ANOVA, performed on latency-to-eat in the novelty-suppressed feeding test, indicates significant main effects of binge-like alcohol exposure (F(1,48) = 10.3, *p* = 0.0024), CBD (F(1,48) = 8.14, *p* = 0.0064), novel environment (F(1,48) = 43.7, *p* < 0.0001) and the interactions between novel environment and CBD (F(1,48) = 5.63, *p* = 0.0217), and between the novel environment and binge-like alcohol exposure (F(1, 48) = 10.3, *p* = 0.0024). CBD administration did not affect latency to eat in the novel arena in the CTRL group (t = 1.73, df = 48.00, *p* > 0.999), while it significantly decreased latency to eat in the novel arena at WD10 (t = 3.50, df = 48.00, *p* = 0.0122) (Figure 6A). The effect of CBD was specific on emotionality, since it did not affect latency to eat in the home cage (CTRL rats: t = 0.0296, df = 48.00, *p* > 0.999; WD10 rats: t = 0.509, df = 46.00, *p* > 0.999). On the other hand, CBD increased rats’ response to a natural reward, in terms of sucrose consumption (main effect of CBD: F(1,24)= 46.01, *p* < 0.0001) (Figure 6A).

When rats were evaluated for coping with the forced swim stress, passive coping observed at WD10 (binge: F(1,24) = 4.69, *p* = 0.040) was counteracted by sub-chronic CBD administration (F(1,24) = 55.5, *p* < 0.001). In detail, within the first 5 min, WD10 rats showed increased immobility with respect to CTRL (t = 3.54, df = 72.0, *p* = 0.046), that was decreased by CBD (t= 6.54, df = 72.0, *p* > 0.001) (Figure 6B). While no difference was observed between CTRL and WD10 rats in the subsequent 5-min time bins (*p* > 0.999), CBD administration decreased the duration of immobility in CTRL rats within the second time bin (*p* < 0.001), and in WD10 rats during the last time bin (*p* = 0.002) (Figure 6B).

As a matter of fact, CBD administration during withdrawal rescued HPA-axis alterations due to binge-like alcohol exposure during adolescence. A two-way ANOVA on data from serum CORT shows a significant main effect of alcohol binging (F(1,24) = 6.696, *p* = 0.0161) and interaction with CBD (F(1,24) = 7.940, *p* = 0.0095). In detail, we observed increased serum CORT levels in WD10 rats (vs CTRL: t = 3.822, df = 24, *p* = 0.0049) that were significantly decreased by CBD (t = 2.980, df = 24, *p* = 0.0390) (Figure 6C). Furthermore, CBD treatment significantly decreased CRH immunopositivity in WD10 rats (F(1, 24)= 40.34, *p* < 0.0001) in both the PVN (t = 3.242, df = 24, *p* = 0.0069) and the NAc (t = 5.740, df = 24, *p* < 0.001) (Figure 6C). In addition, CBD sub-chronic administration during withdrawal counteracted the effects of binge-like alcohol exposure during adolescence on the presynaptic markers of DA signaling in the NAc. In particular, CBD decreased TH-positive immunofluorescence (main effect of binge: F(1,24) = 230.0, *p* < 0.0001; main effect of CBD: F(1,24) = 103.9, *p* < 0.0001) (Figure 6D,G), as well as DAT immunopositivity (main effect of binge: F(1,24) = 16.79, *p* = 0.0004; main effect of CBD: F(1,24) = 262.5, *p* < 0.0001; interaction: F(1,24) = 50.20, *p* < 0.0001). In particular, CBD rescued the high DAT levels observed at WD10 (WD10 vs CTRL: t = 7.907, df = 24, *p* < 0.001; WD10-CBD Vs WD10: t = 16.47, df = 24, *p* < 0.001), with no difference between WD10-CBD and CTRL-CBD levels (t = 2.113, df = 24, *p* = 0.2713) (Figure 6E,G). As to PSD95 immunofluorescence, two-way ANOVA indicates a significant effect of alcohol binging (F(1,24) = 13.65, *p* = 0.0011), CBD (F(1,24) = 13.07, *p* = 0.0014) and their interaction (F(1,24) = 8.259, *p* = 0.0084). Again, CBD treatment counteracted the increase in PSD95 levels observed at WD10 (WD10 vs CTRL: t = 4.645, df = 24, *p* = 0.0006; WD10 vs WD10 + CBD: t= 4.589, df = 24, *p* = 0.0007) (Figure 6F,G). The effects of sub-chronic CBD administration during alcohol withdrawal were also evaluated on the expression of genes related to DA and glutamate signaling in the NAc. Data analysis shows that CBD increased the expression of D1R (F(1,24) = 4.481, *p* = 0.0448) (Figure 6H) and D2R (F(1,24) = 20.73, *p* = 0.0001) (Figure 6H). Notably, sub-chronic CBD administration rescued the effects of binge-like alcohol exposure on Homer 1 expression (F(1,24) = 6.263, *p* = 0.0195; interaction with binge-like alcohol exposure: F(1,24) = 11.09, *p* = 0.0028). In particular, Homer 1 expression was significantly higher in WD10 rats than in CTRL rats (t = 3.194, df = 24, *p* = 0.0234); CBD administration decreased Homer 1 expression in WD10 rats with respect to non-treated counterparts (t = 4.124, df = 24, *p* = 0.0023), up to CTRL levels (t = 1.516, df = 24, *p* = 0.8562), while it did not affect Homer 1 levels in control rats (t = 0.5849, df = 24, *p* > 0.999) (Figure 6H). In addition, CBD administration was able to decrease the expression of Homer 2 (F(1,24) = 4.852, *p* = 0.0375) (Figure 6H), and compensated the effects of binge-like alcohol exposure during adolescence on Arc expression (main effect of CBD: F(1,24) = 20.33, *p* = 0.0001; binge: F(1,24) = 12.09, *p* = 0.0019; interaction: F(1, 24) =11.34, *p* = 0.0026). As a matter of fact, Arc expression was significantly higher at WD10 than in CTRL rats (t = 4.84, df = 24, *p* = 0.0004); CBD did not affect Arc expression in CTRL rats (t = 0.8075, df = 24, *p* > 0.999) while decreased Arc in WD10 rats, with respect to vehicle (t = 5.569, df = 24, *p* < 0.0001), and up to CTRL levels (t = 0.07789, df = 24, *p* > 0.999) (Figure 6H). Similarly, CBD administration counteracted the effects of binge-like alcohol exposure on LIM Kinase 1 expression (main effect of CBD: F(1,24) = 5.529, *p* = 0.0272; effect of the interaction between CBD and binge-like alcohol exposure: F(1,24) = 8.749, *p* = 0.0069). Indeed, CBD decreased LIM Kinase 1 in WD10 rats (t = 3.754, df = 24, *p* = 0.0059) and up to CTRL levels (t = 1.604, df = 24, *p* = 0.7308), whereas it did not affect LIM Kinase 1 expression in CTRL rats (t = 0.4288, df = 24, *p* > 0.999) (Figure 6H). At last, CBD was able to offset the effects of binge-like alcohol exposure during adolescence on FOXP1 expression (main effect of CBD: F(1, 24) = 26.13, *p* < 0.0001; effect of the interaction between CBD and binge-like alcohol exposure: F(1,24) = 25.81, *p* < 0.0001). Notably, sub-chronic administration of CBD decreased FOXP1 expression in WD10 rats (t = 7.207, df = 24.00, *p* < 0.001), up to CTRL levels (t = 0.4416, df = 24.00, *p* > 0.999) and did not modify FOXP1 expression in CTRL rats (t = 0.02228, df = 24.00, *p* > 0.999) (Figure 6H).

## 4. Discussion

The repetitive, cyclic pattern of alcohol binge-drinking to intoxication and withdrawal therefrom is already a trend among adolescents and an important public health concern, as it is largely agreed that the adolescent brain is particularly sensitive to alcohol-induced metaplasticity [60]. The main purpose of this research was, then, to highlight a relationship between a binge-like alcohol exposure in adolescence with alterations in the emotional state and sensitivity to natural positive stimuli in young adult rats. Our hypothesis is that excessive, though intermittent, alcohol intake during adolescence may cause prolonged disarrangement in neural circuits in the NAc, the motivational and integrational hub of the mesocorticolimbic system [61].

Our results show that abstinence following binge-like alcohol exposure during adolescence produces an abnormal reactivity to the environmental challenges, displayed by an increase in anxiety-like behaviour in social- and novel contexts, and impaired reward/aversion processing in adulthood. Several abnormalities occur in stress mediators, DA signaling, excitatory plasticity and synaptic remodeling that may suggest a hyper-sensitiveness of the HPA axis and compensatory mechanisms in the NAc. Intriguingly we found that the administration of CBD, which is known for its potential activity as a regulator of excitatory and inhibitory tone [43] ameliorates most of the behavioural, cellular and molecular alterations induced by alcohol exposure and withdrawal in rats, opening a window towards a putative rescue strategy.

Alcohol produces different outcomes depending on age at exposure (in-utero, adolescence or adulthood) and on time of observation (during intoxication or abstinence_ [52,62,63,64,65,66,67]. In rodents, adolescence encompasses approximately PND 35 to 55, based on the appearance of growth spurts, the pruning of excitatory synapses and behavioural characteristics such as increased peer interaction, play, and exploratory behaviour in the wild (for review see [29]). Here we exposed PND 35 rats to intermittent alcohol administrations in order to reach intoxicating BAC [6], ruling out intra- and inter-individual variability in alcohol intake, typical of self-administration paradigms.

Afterwards, we carried out our evaluations during binge days (BD), early (WD1) and late (WD10) withdrawal. Notably, at these distinct time points, we observed a radical switch in rat behaviour: if at BD, rats did not display significant differences in their emotional state in a social context, as compared to controls, at WD1 and WD10 rats displayed social anxiety-like behaviour, indexed via significant increase in social avoidance and decrease in social investigation, whereas no alterations occurred in locomotor and explorative activity. The social interaction test provides a deep insight into emotional behaviour in laboratory rodents [37]. We employed a modification of the social interaction test, where an experimental animal can freely move towards or away from the compartment of the arena of a nonmanipulated social partner. Here, social avoidance reflects anxiety-like behaviour under social circumstances, as social approach is also directed by motivation [68,69,70]. Reduced motivational salience associated with natural rewarding stimuli is specifically reported in social anxiety disorders and substance abuse, suggesting an involvement of DA-driven reward circuitry [71]. Accordingly, at WD10, rats appeared to suffer from a higher conflict between the anxiogenic novel environment and hunger-induced behaviour in the NSFT as shown by an increase in latency to eat with respect to BD and controls. In this task, rats are challenged by the appeal of a piece of familiar chow in the face of its location in the middle of a novel open arena. Similar to social aversion, hyponeophagia is generally considered an anxiety-like feature as shown by several reports on the anxiogenic effect of alcohol withdrawal in adolescent and adult male rats. Indeed, negative affective behaviour is observed during abstinence in the NSFT and in the forced swim test [72,73,74]. Moreover, the “chronic intermittent ethanol vapor exposure model” triggers negative affective behaviour in male mice in the NSFT after 3 or 5 days of abstinence, and in the marble-burying test at 2 and 10 days of abstinence [75,76,77]. Again, due to the dual component of the rat response—motivation to food and fear of open space—the increase in latency to eat, here observed in abstinent rats at WD10, can result from the intersection of altered motivation/aversion processing and abnormal behavioural reactivity, as a measure of the negative affect stage induced by alcohol withdrawal [78]. On the other hand, when we assessed sensitivity to positive natural stimuli in a neutral environment, at BD we measured a greater sucrose consumption compared to basal levels, which progressively decreased with withdrawal. This result further indicates that natural reward processing is affected by intermittent exposure to intoxicating alcohol levels and forced abstinence.

Interestingly, late withdrawal interfered with rats’ coping strategies to an inescapable stress in the FST, a model able to provide a unique insight into the neural limb of the stress response [79]. When tested at WD10, rats displayed reduction in climbing and increase in immobility during the first 5 min in the water, evening out their behaviour akin to controls’ in the following time bins. This behavioural pattern would indicate disruption in coping strategizing towards adverse environmental conditions, rather than despair, wherein an adaptive prompt response is required by a sudden change of the environment [80]. Indeed, these results are paralleled by an increase in the stress-system activation during late withdrawal, as displayed by higher serum CORT levels and hypothalamic CRH levels at WD10, with respect to controls and BD. Prior work examining the effects of repeated exposure to alcohol in adolescence on the gene expression of critical regulators of stress and anxiety likewise reported increased CRH gene expression in the PVN in human males [81]. On the other hand, in the current experimental model, it seems that the cycle of intoxicating concentrations of alcohol and its absence every other day did not disrupt HPA-axis regulation, at least for as long as the intermittent drinking paradigm continues. Indeed, at WD1 higher serum CORT levels are associated to unmodified CRH expression, suggesting the maintenance of a regular negative feedback in the hypothalamus. Once alcohol administration is suspended, an alteration in the balance between HPA drive and feedback is emphasized, as shown by the concurrent increase in CRH and CORT, which highlights a hypersensitivity of the brain’s stress response [78]. This result is in agreement with previous evidence demonstrating that binge-like alcohol exposure during pubertal development increases both circulating plasma CORT levels and CRH mRNA expression in the PVN, pointing to the active role played by binge alcohol-drinking in the disruption of normal glucocorticoid negative feedback pathways [81,82]. CRH expression is also increased in the NAc, both at WD1 and WD10. Augmented extra-hypothalamic CRH expression has been correlated with the occurrence of negative affective behaviour during alcohol withdrawal [78]. Thus, the emotional dysregulation occurring during the withdrawal days here reported, might result from a between-system—rather than a within-system—interference, in which CRH may act as a contextual signal, gating the NAc neural maladaptation associated to binge-like alcohol exposure [83]. Indeed, several reports show that CRH affects mesolimbic DA-ergic neurones by producing an increase in their functional state, in terms of DA synthesis, firing, release and reuptake [84,85]; it follows, then, that NAc DA is involved in the shaping of stress reactivity and motivated behaviour [86,87]. It is not surprising that the high activity and sensitization of the DA-ergic system occurring during adolescence may respond dynamically to alcohol stimulation in the repeating cycle of binging doses and forced abstinence in an intermittent paradigm. When DA signaling and synaptic plasticity in the NAc are analysed, a profound change in the temporal dynamics of the components of the striatal DA system is displayed. At BD, if presynaptic DA synthesis, measured by TH levels, does not differ from controls, the synaptic availability of the neurotransmitter appears to be increased, since DAT expression decreases. On the postsynaptic side, a distinguished pattern of expression regards D1 and D2 receptors’ mRNA, with D1R being overexpressed and D2R displaying a significant reduction, as compared with controls. This peculiar feature would suggest a higher responsivity of the reward system to positive stimuli, as corroborated by a positive correlation between sucrose consumption and D1R expression. Indeed, the inhibition of DAT activity by nomifensine does increase DA availability in the NAc and is reported to increase sucrose intake, according to a D1R-mediated effect [88,89]. Notably at BD, in accordance with the behavioural patterns observed in response to social and novel contexts, the indices of synaptic plasticity taken into consideration in this study do not show significant alterations, compared with controls.

The intermittence of the binge-like alcohol exposure does not affect DA synthesis, whereas DAT expression is significantly augmented with respect to BD, producing a relative reduction in DA synaptic availability at WD1. This evidence is an accordance with several reports showing a hypo-DAergic input during acute withdrawal after chronic alcohol exposure [15,16,90]. On the striatal post-synaptic side, we observed a progressive switch in D1R- and D2R-mRNA expression, with D1R decreasing and D2R increasing during withdrawal. Although currently it is not possible to prove a direct causal relationship, the peculiar dynamics of the striatal components of the DA system may contribute to the increase in social avoidance observed at WD1; indeed, a reduced DA-ergic input corresponds to an increase in social avoidance [68]. The framework changes at WD10, when the increase in DA synthesis is associated with an increase in DA reuptake from the synaptic cleft, displaying a decrease in D1R and an increase in D2R expression. As a matter of fact, the primary mechanism controlling the concentration of extracellular DA is the selective uptake by the high-affinity DA transporter [85], implying that the increase in DA-ergic synthesis measured at WD10 might not correspond to increased DA availability in the synaptic cleft. In accordance, our results suggest a limitation in the duration and strength of the elicited DA-ergic signaling and in the behavioural outcomes of the event during prolonged abstinence, as shown by an altered response to natural stimuli, an increase in anxiety-like behaviour, and a dysfunctional coping with stress. Besides, the reduced D1R- and the increased D2R-expression levels in the NAc indicate that both receptors are sensitive to perturbation after withdrawal from binge-like alcohol exposure during adolescence.

Stress-related modifications in limbic synaptic plasticity converge on the NAc as a significant locus of integration and, in particular, on glutamatergic signaling [91]. Therefore, it is not unexpected that the hyperactivity of the HPA axis and the NAc over-signaling CRH, here reported during withdrawal, were associated with altered indices of glutamatergic transmission. In particular, an increase in the excitatory scaffolding proteins PSD95 and Homer was measured, indicating an abnormal synaptic strength of glutamate transmission in the MSNs [92].

Abnormal levels of extracellular glutamate in the NAc have been reported in different models of binge-like drinking [93,94,95,96]. A recent study employing proton magnetic resonance spectroscopy has reported elevated glutamate concentration in the NAc of recently detoxified patients [97], confirming the plethora of findings from the preclinical models of alcohol withdrawal [98]. Moreover, recent data show that glutamate synergistic activation of NMDA and mGLU5 receptors strengthen the synapse-to-nucleus communication via an increase in the phosphorylation of extracellular signal-regulated protein kinase (ERK), known as a mediator of inducible gene expression [99]. Interestingly, the integral regulation of ERK is dependent on the cross-talk between NMDA receptor-associated synaptic adaptor protein PSD95 and the mGluR5-linked adaptor protein Homer [100]. Therefore, it is evocative to hypothesize that at WD10 the (hyper-)activation of glutamate transmission is associated with the synergistic over-expression of PSD95 and Homer scaffolding which, in turn, promote a new asset in the effector systems regulating the expression of specific markers of synaptic remodeling. Within the NAc, alcohol induces neuroplastic changes by remodeling dendritic spines [15]. In accordance, we report a selective increase in players of neuronal activity such as Arc, an immediate early gene that is involved in neuroplasticity within the soma and dendrites [101], and of cytoskeleton dynamics such as LIMK1 at late withdrawal [102]. Seminal studies have revealed that NMDAR stimulation appears necessary for the synthesis and targeting of Arc mRNA to “on-line” synaptic regions, within dendrites, where stressful conditions can increase Arc expression and lead to the consolidation of neuroplasticity [103,104]. Therefore, we suggest that the enrichment in NAc Arc mRNA expression can represent not only a molecular marker of abnormal excitatory neuroplasticity, but also of social- and novelty-related anxiety-like behaviour during alcohol withdrawal [105]. LIM Kinase 1 is highly expressed in neurones, where it regulates actin dynamics. Overexpression of LIM Kinase 1 in cultured cells results in actin stabilization as filamentous actin, which supports the spine shape and drives the postsynaptic signaling pathway to maintain spine stability and the dynamic response to many extracellular cues [102,106].

Overall, the current findings suggest an intersection between modifications in excitatory postsynaptic transmission and cytoskeleton-related activity, setting a new architecture in the MSNs in the NAc. This picture is enriched by the finding of an upregulated expression of FOXP1 at WD10 with respect to the other time points of observation. Among the different transcriptional factors implicated in the development and maturation of neuronal cells, FOXP1 is particularly abundant in the striatum compared to the rest of the brain [107]. Here it co-localises with markers of MSNs and is tightly controlled by neuronal activity via synapse-to-nucleus signaling, mediated by NMDAR [108]. To our knowledge, no report is available on the functional significance of overexpressed FOXP1 in the NAc. However, based on the current findings, we hypothesize that the overexpression of FOXP1 at WD10 might further affect the maturation of intrinsic excitability of MSNs [109], thus regulating cellular composition and neurochemical architecture of the striatal circuitry during alcohol withdrawal metaplasticity [110].

In the attempt to find a sound interpretation of all these pieces and put the puzzle together, we explored the main axes of variance within the data set by PCA. The analysis identified the behavioural patterns related to social and environmental anxiety and motivational processing, and the neuroendocrine parameters and concurrent DA and glutamate neuroplasticity in the NAc as key variables contributing to the vector that, more than others, captures the data’s orientation. Interestingly, we observed that the features associated with sensitivity to positive natural stimuli and D1R expression in the NAc display an opposite sign with respect to features of anxiety-related behaviours, suggesting an inverse relationship between them. The divergence of social investigation- and play-related vectors, with respect to sucrose consumption and D1R expression, suggests the involvement of distinct neural systems in the genesis of the two behavioural dimensions, which appear discretely affected by binge-like alcohol exposure during adolescence [33,39,111]. In addition, the correlation matrix revealed that the abnormal behavioural response to social and novel contexts is significantly linked to the intersecting alterations in both stress response and in pre- and postsynaptic neuroplasticity in the NAc. Interestingly, a significant association between FOXP1 expression and all the behavioural readouts produced by binge-like alcohol exposure in adolescence was highlighted. Although, at this stage, it is difficult to draw a conclusion, this evidence suggests an intriguing though ventured perspective, pointing to FOXP1 as a stabilizing factor of the maladaptive synaptic remodeling associated with the peculiar behavioural phenotype observed at late withdrawal. Intriguingly, we also show that metaplasticity, resulting from chronic intermittent-alcohol withdrawal, is hindered by sub-chronic administrations of CBD. Indeed, CBD was able to counteract the behavioural alterations induced by late alcohol withdrawal, restored an adaptive response of the HPA axis, fine-tuned accumbal CRH levels, and discretely rectified indices of pre-and postsynaptic neuroplasticity. In particular, CBD increased social investigation and decreased social avoidance, which were not related to changes in motor activity, since no difference was found in exploratory activity between the experimental and control groups, in line with previous observations [112,113]. Accordingly, CBD treatment during alcohol withdrawal in rats elicited anxiolytic and pro-active effects in NSFT and FST, respectively, further supporting CBD’s potential anti-stress properties. This is in agreement with the anxiolytic-like effect in NSFT and in EPM described by Fogaça and colleagues [114], which seems to involve a potentiation in endocannabinoid’s signaling through CB1 receptors [115]. The positive effects of CBD in the FST are confirmed in other studies, after both acute and chronic treatment at different doses (30-, 100- and 200 mg/kg, i.p., [116,117,118]. Furthemore, pretreatment with WAY100635 inhibited CBD effects, suggesting the involvement of 5-HT1A receptors [119]. Adaptive changes in pre- and postsynaptic 5-HT1A receptor functionality were also found after chronic CBD (50 mg/kg/daily/3 days + 10 mg/kg/daily/11 days) administration [119].

Besides thes, in our experimental conditions, CBD was able to bring CRH, and CORT signaling back to control levels. It is already known that CBD, at different doses, decreases the HPA-axis response under stress condition, as shown by lower CRH mRNA levels in PVN after 20 min of acute restrain stress [120]. Moreover, CBD reduces cortisol levels and anxiety in drug-abstinent patients, likely facilitating endocannabinoid signaling on limbic neural circuits engaged during negative emotional processes [44,121]. On the other hand, CBD is reported to modulate reward processing in the mesolimbic system [122], interfering with DA synthesis and postsynaptic responses [123,124]. In accordance with CBD-induced reduction in TH gene expression in the VTA of alcohol-drinking rats [47], our data show lower TH activity, accompanied by decremental levels of DAT immunoreactivity in the NAc, independently of alcohol exposure. At the same time, a significant increase in D1 and D2R expression was recorded after CBD treatment, in both WD10 and control rats, suggesting that the changes at the post-synaptic level aim at restoring DA signaling in the NAc. Notably, CBD-induced modulation of VTA DA activity may also occur through regulatory action on glutamatergic projections in the NAc [125]. Consistently, we observed a down regulation in the expression of the glutamate-associated proteins here analysed, which suggests a functional remodeling of the postsynaptic side. In this new architecture, it is compelling to involve even FOXP1 as an index of immature plasticity, which is upregulated during withdrawal and restored to normal levels by CBD. Indeed, evidence supports the role of CBD in modulating intracellular pathways directly related to synaptic remodeling. In particular, a synapse-restoring effect was highlighted in chronically stressed mice, where repeated CBD treatment promoted an increase in PSD95 expression, synapsyn I/IIi and synaptophysin [126].

## 5. Conclusions

Overall, the current evidence evokes a new frame of reference, where, during withdrawal from binge-like alcohol exposure, an anxiety-prone phenotype, characterized by an upregulated response to stress plus reduced sensitivity of the reward system, is associated with dysfunctional synaptic remodeling, probably via the interplay between the augmented CRH expression and the limited availability of accumbal DA [127]. In our opinion, it is unworkable to dissect and consistently interpret all the factors playing in the CBD-associated remake of the MSN synapse, which is here reported for the first time. The potential rescue activity of CBD, however, certainly deserves further investigation, also in the light of the high spread of light cannabis use and the need for scientific evidence supporting its rationale in pharmacotherapy.

## Figures and Tables

**Table 1:** Primers employed in qRT-PCR experiments.

Gene Name	Primer Sequence
Gapdh	GTTTGTGATGGGTGTGAACC (Forward)
	CTTCTGAGTGGCAGTGATG (Reverse)
Dopamine Receptor 1	ACAGATGCATTGTTGATGAC (Forward)
	TGCTAGTACAAATGGAGAGG (Reverse)
Dopamine Receptor 2	TTAACATCGTCTCTCTTCCA (Forward)
	ACAGGTATAGTGATGTTACA (Reverse)
Homer 1	CTTCACAGGAATCAGCAGGAG (Forward)
	GTCCCATTGATACTTTCTGGTG (Reverse)
Homer 2	AAGATCGCTTTGACACAGAG (Forward)
	CTCGCTGCACTGTTCTTCCA (Reverse)
Activity-Regulated Cytoskeleton Associated Protein	ACAGAGGATGAGACTGAGGCAC (Forward) TATTCAGGCTGGGTCCTGTCAC (Reverse)
LIM Domain Kinase 1	ATGAGGTTGACGCTACTTTGTTG (Forward)
	CTACACTCGCAGCACCTGAA (Reverse)
Forkhead Box p1	CACGTGGAAGAATGCAGTGC (Forward)
	GCCTGTAAAGCTGCATTGAG (Reverse)

## Data Availability

Data supporting reported results are available on request from the corresponding author.
